# Evidence for a direct link between PAD4-mediated citrullination and the oxidative burst in human neutrophils

**DOI:** 10.1038/s41598-018-33385-z

**Published:** 2018-10-15

**Authors:** Yebin Zhou, Ling-Ling An, Raghothama Chaerkady, Nanette Mittereder, Lori Clarke, Taylor S. Cohen, Bo Chen, Sonja Hess, Gary P. Sims, Tomas Mustelin

**Affiliations:** 1 Department of Respiratory, Inflammation, and Autoimmunity, MedImmune LLC, One MedImmune Way, Gaithersburg, Maryland 20878 United States; 2 Antibody Discovery and Protein Engineering, MedImmune LLC, One MedImmune Way, Gaithersburg, Maryland 20878 United States; 3 Department of Microbial Sciences, MedImmune LLC, One MedImmune Way, Gaithersburg, Maryland 20878 United States; 4 Present Address: Division of Rheumatology, School of Medicine, University of Washington, 750 Republican Street, Seattle, WA98109 United States

## Abstract

Neutrophils are critical for the defense against pathogens, in part through the extrusion of extracellular DNA traps, phagocytosis, and the production of reactive oxygen species. Neutrophils may also play an important role in the pathogenesis of rheumatoid arthritis (RA) through the activation of protein arginine deiminases (PADs) that citrullinate proteins that subsequently act as autoantigens. We report that PAD4 is physically associated with the cytosolic subunits of the oxidative burst machinery, p47^phox^ (also known as neutrophil cytosol factor 1, NCF1) and p67^phox^ (NCF2). Activation of PAD4 by membranolytic insults that result in high levels of intracellular calcium (higher than physiological neutrophil activation) leads to rapid citrullination of p47^phox^/NCF1 and p67^phox^/NCF2, as well as their dissociation from PAD4. This dissociation prevents the assembly of an active NADPH oxidase complex and an oxidative burst in neutrophils stimulated by phorbol-ester or immune complexes. In further support of a substrate-to-inactive enzyme interaction, small-molecule PAD inhibitors also disrupt the PAD4-NCF complex and reduce oxidase activation and phagocytic killing of *Staphylococcus aureus*. This novel role of PAD4 in the regulation of neutrophil physiology suggests that targeting PAD4 with active site inhibitors for the treatment of RA may have a broader impact on neutrophil biology than just inhibition of citrullination.

## Introduction

Neutrophils are critically important for our defense against bacterial pathogens^1,2^. They are among the very first responders to sites of tissue injury and bacterial infection, where they use a large repertoire of receptors for bacterial and pro-inflammatory stimuli to trigger a range of responses that include the extrusion of neutrophil extracellular traps (NETs)^3–7^, phagocytosis^1,8^, reactive oxygen species (ROS) production^9–11^, and the secretion of numerous proteases and pro-inflammatory mediators^12,13^.

Neutrophils are also attracting increasing attention as likely culprits in human diseases such as rheumatoid arthritis (RA)^14–16^ and systemic lupus erythematosus (SLE)^17–20^. In RA, neutrophils accumulate in the synovial fluid in the early stages of the disease^21,22^ and appear to be responsible for the increase in citrullination of numerous proteins observed in the synovial fluid of inflamed joints^22–25^. Approximately 70% of RA patients test positive for antibodies against citrullinated cyclic peptides (anti-CCPs)^26,27^, and have antibodies reactive with specific citrullinated epitopes in proteins such as filaggrin, fibrinogen, vimentin^28^, α-enolase, and histones^14,15^. Further support for an important role of protein citrullination in RA pathogenesis comes from genetic association studies, which implicated first *PADI4*^29–33^ and later also *PADI2*^34^ in the susceptibility to RA. These genes encode for the Ca^2+^-dependent protein arginine deiminases (PAD) 4 and 2, respectively, which catalyze the conversion of arginine to citrulline in protein substrates^35–38^.

Neutrophils express both PAD2 and PAD4^39,40^. The former is found in many cell types, while the latter is more restricted to hematopoietic cells with highest levels in neutrophils. PAD4 contains a putative nuclear localization sequence^41^, but resides mostly in cytosolic structures in resting neutrophils^40^. PAD4 can also enter the nucleus to citrullinate histones^42^ and transcription factors^43^, and thereby participate in the epigenetic regulation of gene expression^44^ and stem cell differentiation^43^. Histone citrullination has also been implicated in the formation of NETs^45–48^, a complex process driven by multiple signaling pathways and that depends on ROS production (at least for some stimuli such as phorbol 12-myristate 13-acetate (PMA)) and involves a large transcriptional program^49^. Extruded NETs serve to trap bacteria^3^ and aid in the subsequent phagocytosis and killing process. NET formation can be induced by many stimuli^48^, including PMA, immune complexes, and live bacteria. Dysregulation of NET extrusion has been proposed to play a role in the pathogenesis of autoimmune conditions^7,14,16–19,50^, although it remains unclear if extracellular DNA and histones found in kidneys of patients with anti-neutrophil cytoplasmic antibody (ANCA)-associated vasculitis^17^, or in the circulation of patients with SLE^18,19^, originate only from NETs or also from other forms of tissue damage and cell death. In addition, a recent paper has shown that PAD4 can citrullinate NF-κB p65 to promote its nuclear translocation^51^.

We have found that two cytosolic subunits of nicotinamide adenine dinucleotide phosphate (NADPH) oxidase, p47^phox^ (also known as neutrophil cytosol factor 1, NCF1) and p67^phox^ (neutrophil cytosol factor 2, NCF2) are citrullinated in primary human neutrophils upon activation of PADs by membranolytic stimuli. Furthermore, NCF1 has been found to be citrullinated in synovial fluid from RA patients^24,25^. NADPH oxidase is the main ROS machinery in neutrophils, and its activation is key in bacterial killing^8–11^. We provide evidence that PAD4 in neutrophils associates with NCF1 and NCF2, translocates with them during NADPH oxidase activation, and is important in ROS production and bacterial killing. This association requires PAD4 to remain inactive (*i.e*., a non-enzymatic function); conversely PAD4-mediated citrullination of NCF proteins dissociates the complexes and impairs ROS generation. Disruption of the NADPH oxidase-PAD4 complex by other means also inhibits ROS production, leading to reduced killing of bacteria.

## Results

### Citrullination of cytosolic subunits of NADPH oxidase

Neutrophils subjected to membranolytic agents that cause a rapid and lethal influx of calcium undergo a rapid citrullination of multiple cellular proteins^23,52,53^. This response was termed ‘lethal hypercitrullination’ by Konig and Andrade^53^ and results in intracellular Ca^2+^ concentrations that exceed the physiological receptor-triggered Ca^2+^ mobilization response by 2–3 orders of magnitude. We used LC-MS/MS to identify a set of proteins that became citrullinated after treatment of human neutrophils with 1 µM ionomycin (in medium with 2 mM Ca^2+^) or perforin (data not shown). Several of these proteins, including the cytosolic subunits p47^phox^ (NCF1) and p67^phox^ (NCF2), were also reported to be citrullinated in the synovial fluid of a RA patient in a recent paper^24^. We found p47^phox^/NCF1 to be citrullinated at 6 arginine residues (R7, R70, R85, R90, R335, R336) and p67^phox^/NCF2 at 4 (R184, R188, R238, R328) (Table 1, Fig. S1). None of these sites were detected in untreated neutrophils. These results suggest that NADPH oxidase subunits are citrullinated during calcium influx driven PAD activation. Interestingly, the citrullinated residues are located in the PX domain and in the middle of the phosphorylated tail of NCF1 and on either side of the N-terminal SH3 domain of NCF2. The locations are in regions known to be involved in protein-protein interactions. In addition, R90 in NCF1 is also the site of a polymorphism, R90H, associated with systemic lupus erythematosus^54–57^.

**Table 1:** NCF citrullination in ionomycin/calcium treated neutrophils.

Annotated Peptide Sequence	Modifications	Donors (Total =4)	Protein
WFDGQr(cit)AAENR	R85(Citrulline)	4	NCF1
WFDGQr(cit)AAENr(cit)QGTLTEYcSTLMSLPTK	R85(Citrulline) R90(Citrulline);	1	NCF1
EMFPIEAGAINPENr(cit)IIPHLPAPK	R70(Citrulline	1	NCF1
FLQqr(cit)r(cit)RqARPGPQSPGSPLEEER	Q334(Deamidated) R335(Citrulline) R336(Citrulline) Q338(Deamidated)	1	NCF1
GDTFIr(cit)HIALLGFEK	R7(Citrulline);	1	NCF1
APGr(cit)PQLSPGQK	R328(Citrulline)	2	NCF2
LFr(cit)PNEr(cit)QVAQLAK	R184(Citrulline); R188(Citrulline)	1	NCF2
TPEIFr(cit)ALEGEAHR	R238(Citrulline)	2	NCF2

### Co-immunoprecipitation of PAD4 with cytosolic subunits of NADPH oxidase

**Figure 1:**
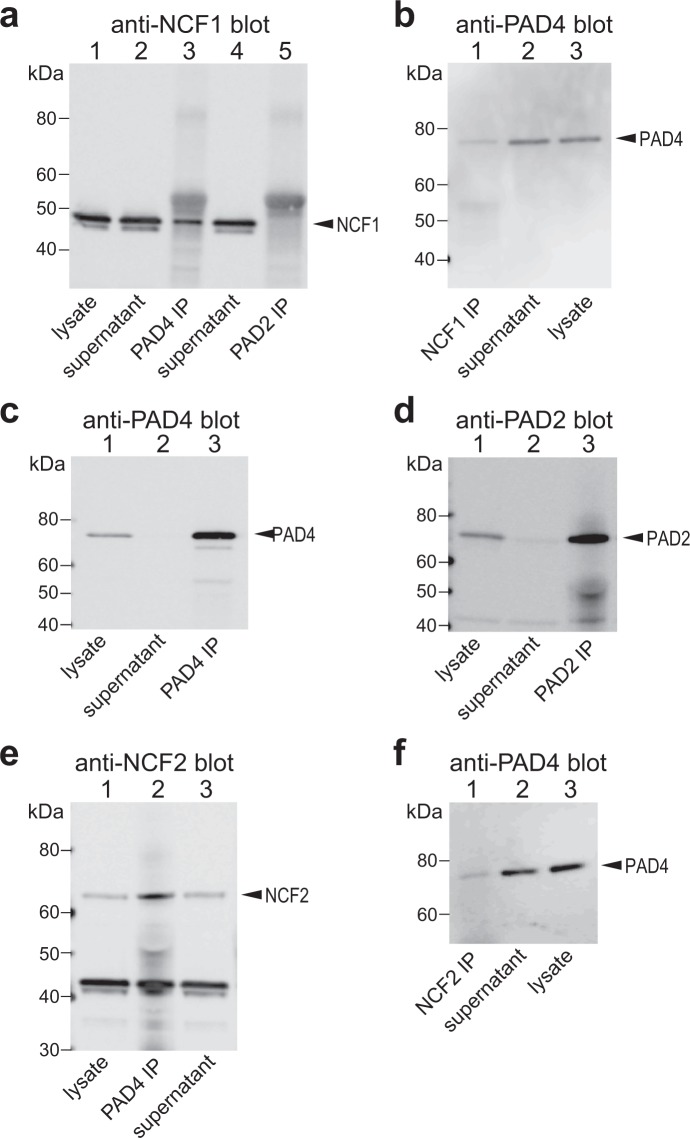
Co-immunoprecipitation of PAD4 with the NCF1 and NCF2 subunits of NADPH oxidase. (**a**) Anti-NCF1 immunoblot of total lysate from resting neutrophils (lane 1), supernatant after immunoprecipitation with anti-PAD4 (lane 2), immunoprecipitates with anti-PAD4 (lane 3), supernatant after immunoprecipitation with anti-PAD2 (lane 4), or immunoprecipitates with anti-PAD2 antibodies (lane 5). (**b**) Anti-PAD4 immunoblot of anti-NCF1 immunoprecipitates (lane 1), supernatant after immunoprecipitation with anti-NCF1 (lane 2), or total lysate from resting neutrophils (lane 3). (**c**) Anti-PAD4 immunoblot of the samples in lanes 1–3 in panel a. (**d**) Anti-PAD2 immunoblot the samples in lanes 1, 4, and 5 in panel a. (**e**) NCF2 western blot of lysate from resting neutrophils (lane 1), anti-PAD4 immunoprecipitates, or the supernatant after immunoprecipitation (lane 3). (**f**) Anti-PAD4 immunoblot of anti-NCF2 immunoprecipitates (lane 1), supernatant after immunoprecipitation with anti-NCF21 (lane 2), or total lysate from resting neutrophils (lane 3). The data represent the results of five independent experiments.

As binding between an enzyme and its substrate is sometimes more stable, we tested if PAD4 and p47^phox^/NCF1 or p67^phox^/NCF2 could be co-immunoprecipitated. PAD4 or PAD2 were immunoprecipitated from untreated neutrophils from healthy donors and immunoblotted with specific antibodies to the NCF proteins. As shown in Fig. 1, p47^phox^/NCF1 and p67^phox^/NCF2 were both readily detected in anti-PAD4, but not in anti-PAD2, immunoprecipitates. The reverse experiment was also true: immunoprecipitates obtained with antibodies to NCF1 and NCF2 contained immunoreactive PAD4 (Fig. 1).

### Co-localization of PAD4 with NCF1 in intact neutrophils

**Figure 2:**
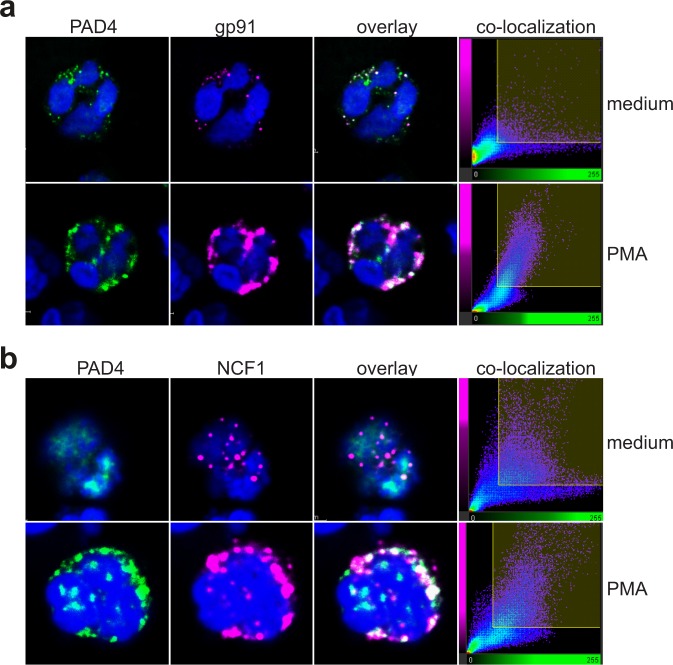
Co-localization of PAD4 with gp91 and NCF1 in resting or activated neutrophils. (**a**) Confocal images of resting neutrophils (upper panels) or neutrophils stimulated with 25 nM PMA (lower panels) stained with anti-PAD4 (green) or anti-gp91 (magenta). (**b**) Confocal images of resting neutrophils (upper panels) or neutrophils stimulated with 25 nM PMA (lower panels) stained with anti-PAD4 (green) or anti-NCF1 (magenta). The fourth panels show the co-location of pixels of the two colors. The data represent the results of three independent experiments.

**Figure 3:**
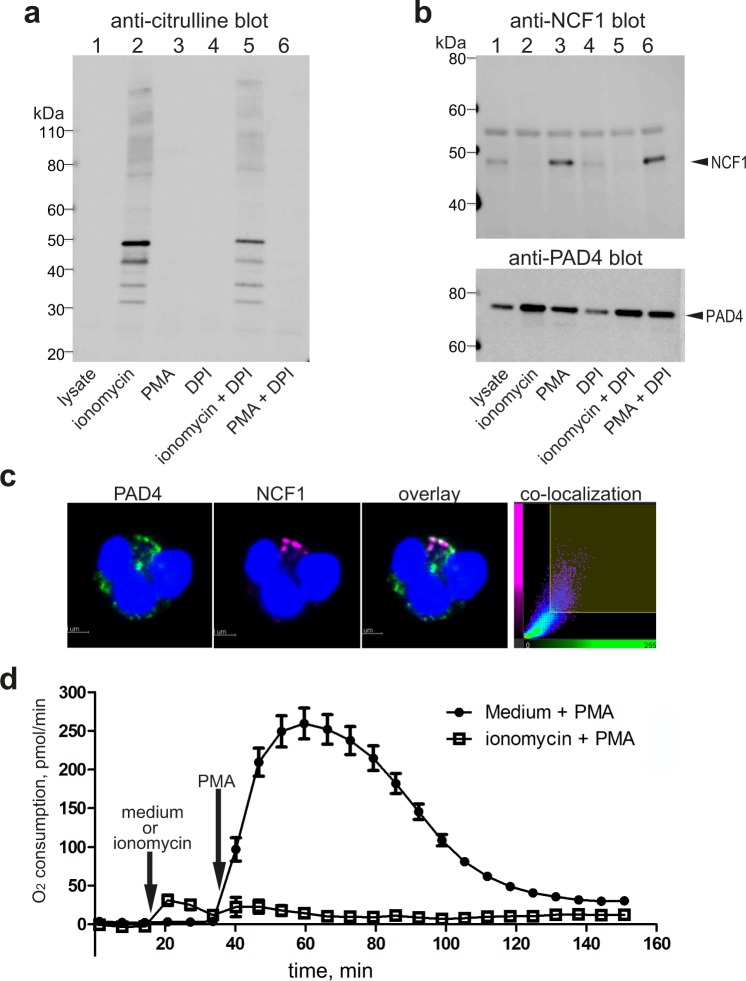
Calcium-induced PAD4 activation dissociates PAD4-NCF1 complexes and inhibits ROS generation. (**a**) Citrullination of neutrophil proteins detected by anti-citrulline antibody F95 in cells stimulated with 1 µM ionomycin (in medium with 2 mM Ca^2+^), 100 nM PMA, or 10 µM DPI, as indicated. (**b**) Upper panel, anti-NCF1 immunoblot of anti-PAD4 immunoprecipitates from resting neutrophils (lane 1), or cells treated with the indicated stimuli. Lower panel, anti-PAD4 immunoblot of the same samples. (**c**) Confocal images of neutrophils treated with 1 µM ionomycin (in medium with 2 mM Ca^2+^) and then stimulated with 25 nM PMA and stained with anti-PAD4 (green) or anti-NCF1 (magenta). The last panel shows the co-location of pixels of the two colors. (**d**) Oxygen consumption as a measure of ROS generation by neutrophil stimulated by 100 nM PMA, first treated medium alone or with 1 µM ionomycin, as indicated. The data represent the results of three independent experiments.

Activation of the oxidative burst machinery is known to involve the translocation of the cytosolic subunits p47^phox^/NCF1 and p67^phox^/NCF2 to the phagosome or plasma membrane where the NADPH oxidase gp91 and p22 reside^8–11^. To visualize these events and to determine how PAD4 behaves during PMA-induced activation of the oxidase, we stained NADPH oxidase in freshly isolated neutrophils. As we reported recently^40^, PAD4 was found in resting neutrophils in a set of cytoplasmic granular structures with some nuclear localization in ~1% of the cells (Fig. 2a, first panel). In the same cells, gp91 was also found in scattered structures (second panel), which overlapped only to a low extent with PAD4 (third and fourth panels). However, upon treatment of the cells with PMA, both proteins became much more prominently co-localized in larger vesicular structures (Fig. 2a, second row). Very similar results were observed with PAD4 and NCF1: some co-localization in resting neutrophils and a pronounced co-localization in cells treated with phorbol ester (Fig. 2b). In agreement with this, we found that co-immunoprecipitation also improved after PMA treatment (see Fig. 3).

### PAD4 activation by calcium influx disrupts NCF1-PAD4 interaction

To examine whether ionomycin-induced citrullination of NCF1 and 2 affects their association with PAD4, freshly isolated neutrophils were treated with ionomycin, phorbol ester (which activates NADPH oxidase) alone or together with diphenyleneiodonium (DPI) which blocks NADPH oxidase. Figure 3a shows the citrullination of cellular proteins after these stimuli. When anti-PAD4 immunoprecipitates from these cells were immunoblotted with anti-NCF1 antibodies, it was clear that ionomycin-treatment abolished any detectable association, while PMA treatment increased it (Fig. 3b). Consistent with these results, analysis of anti-PAD4 immunoprecipitates by mass spectrometry detected unmodified (i.e. not citrullinated) peptides of NCF1 in samples from resting or PMA-treated cells, but not from ionomycin-treated cells (Table S1). The presence of unmodified peptides from NCF1 and 2, but a complete absence of citrullinated peptides, in anti-PAD4 immunoprecipitates from neutrophils supports the notion that citrullination disrupts the association with PAD4.

By immunofluorescence, we also note that PAD4 and NCF1 are poorly co-localized in ionomycin pretreated cells after PMA stimulation (Fig. 3c). There also was no detectable relocation to the plasma membrane or larger cytosolic vesicles.

### Calcium influx-driven citrullination also blocks ROS generation

As a first test of a possible functional importance of PAD4-NCF association and/or NCF citrullination, we measured the PMA-induced oxidative burst of neutrophils pretreated with ionomycin or medium alone. When stimulated with PMA, NADPH oxidase activation greatly increased neutrophil consumption of oxygen (Fig. 3d). However, when the neutrophils were first treated with ionomycin, PMA-induced ROS generation was completely inhibited (Fig. 3d). These results suggest that dissociation from PAD4, NCF citrullination, or unrelated ionomycin-induced events impair the oxidative burst in neutrophils.

### Small-molecule inhibitors of PAD4 disrupt NCF association and inhibit ROS generation

**Figure 4:**
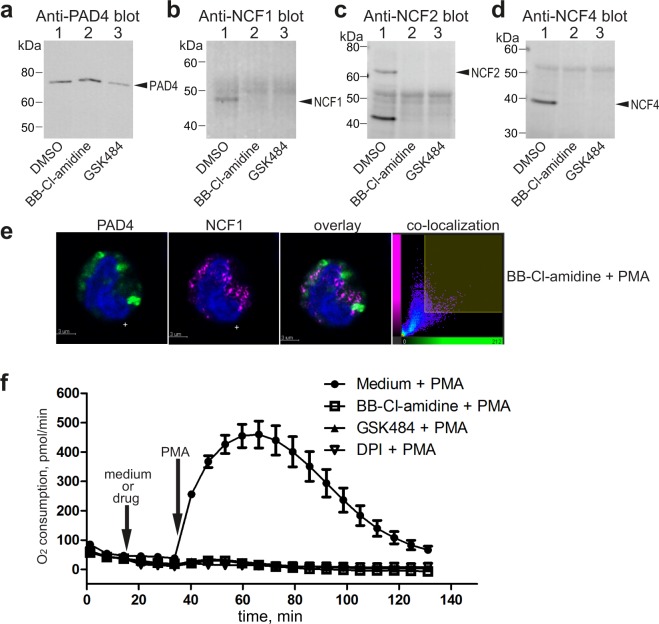
Small-molecule inhibitors of PAD4 disrupt PAD4-NCF association and inhibit ROS generation. (**a**) Anti-PAD4 immunoblot of anti-PAD4 immunoprecipitates from neutrophils treated with DMSO solvent control or the indicated inhibitors. (**b**) Anti-NCF1 immunoblot of the same samples. (**c**) Anti-NCF2 immunoblot of the same samples. (**d**) Anti-NCF4 immunoblot of the same samples. (**e**) Confocal images of neutrophils pretreated with BB-Cl-amidine and then stimulated with 100 nM PMA, stained with anti-PAD4 (green) or anti-NCF1 (magenta). The last panel shows the co-location of pixels of the two colors. (**f**) Oxygen consumption by neutrophils pre-treated with medium alone, 20 µM BB-Cl-amidine, 25 µM GSK484, or 10 µM DPI, as indicated, and then stimulated with 100 nM PMA. The data represent the results of five independent experiments.

Since the PAD4-NCF interaction occurred in the absence of citrullination, and appears to be disrupted by citrullination of NCF, we hypothesized that this interaction may involve the substrate-binding catalytic site of PAD4. Using two commercially available small-molecule PAD4 inhibitors, BB-Cl-amidine and GSK484 that bind to the active site of PAD4^46,58^, we found that NCF proteins can no longer be co-immunoprecipitated with PAD4 after a brief treatment of neutrophils with these drugs (Fig. 4a–d). The two inhibitors also reduced the co-immunoprecipitation of gp91 with anti-NCF1 antibodies from PMA-treated neutrophils (Fig. S2). By immunofluorescence, we also note that PAD4 and NCF1 are poorly co-localized in BB-Cl-amidine pretreated cells after PMA stimulation (Fig. 4e). There also was no detectable relocation to the plasma membrane or larger cytosolic vesicles. Furthermore, both drugs blocked PMA-stimulated ROS generation in neutrophils similarly to NADPH oxidase inhibitor DPI (Fig. 4f). Taken together, these data suggest that PAD4 association is important for NADPH oxidase activation in neutrophils.

### PAD4 knockdown reduces and PAD4 overexpression increases ROS in HL60 cells

**Figure 5:**
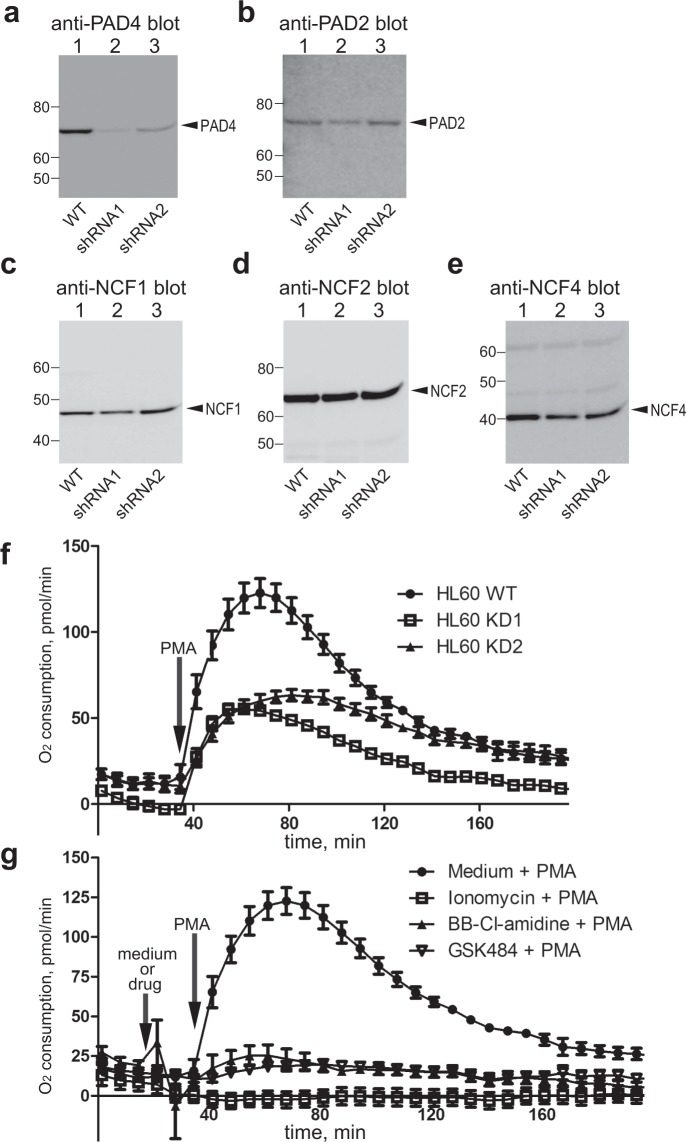
Reduced ROS generation in PAD4 knock-down HL60 cells. (**a**) Anti-PAD4 immunoblot of total cell lysates from HL-60 cells treated with medium alone (lane 1), or the two shRNAs targeting PAD4 (lanes 2 and 3). (**b**) Anti-PAD2 immunoblot of the same lysates. **c)** Anti-NCF1 immunoblot of the same lysates. (**d**) Anti-NCF2 immunoblot of the same lysates. (**e**) Anti-NCF4 immunoblot of the same lysates. (**f**) Oxygen consumption by parental HL-60 cells or HL-60 cells pretreated for 7 days with 2 different shRNAs targeting PAD4, as indicated, stimulated with 100 nM PMA. (**g**) Oxygen consumption by HL-60 pre-treated with medium alone, 1 µM ionomycin, 20 µM BB-Cl-amidine, or 25 µM GSK484, and then stimulated with 100 nM PMA. The data are representative of three independent experiments.

Since small-molecule inhibitors may also have unintended (unknown) effects, we examined the role of PAD4 in NADPH oxidase-mediated ROS generation by shRNA-mediated knock-down of PAD4 protein in the promyelocytic cell line HL60, which can be differentiated to a neutrophil-like phenotype with 0.85% N,N-dimethylformamide. During this differentiation, PAD4 expression increases to a level comparable to that in human neutrophils. Two independent shRNAs resulted in approximately 95 and 80% knock-down of PAD4 proteins, whereas PAD2 and NCFs protein expression were unaffected (Fig. 5a–e). ROS generation was also reduced by approximately 50% in the PAD4-knockdown cells compared to control cells (Fig. 5f). In contrast, PMA-induced ROS generation was increased when PAD4 was overexpressed in HL60 cells (Fig. S3). Like neutrophils, differentiated HL60 produced high levels of ROS after PMA stimulation, and ROS generation was inhibited by BB-Cl-amidine, GSK484, and ionomycin/calcium treatment (Fig. 5g). These results further indicate PAD4 protein plays a permissive role in NADPH oxidase-mediated ROS generation.

### Killing of phagocytosed Staphylococci is reduced by PAD4 inhibitors

**Figure 6:**
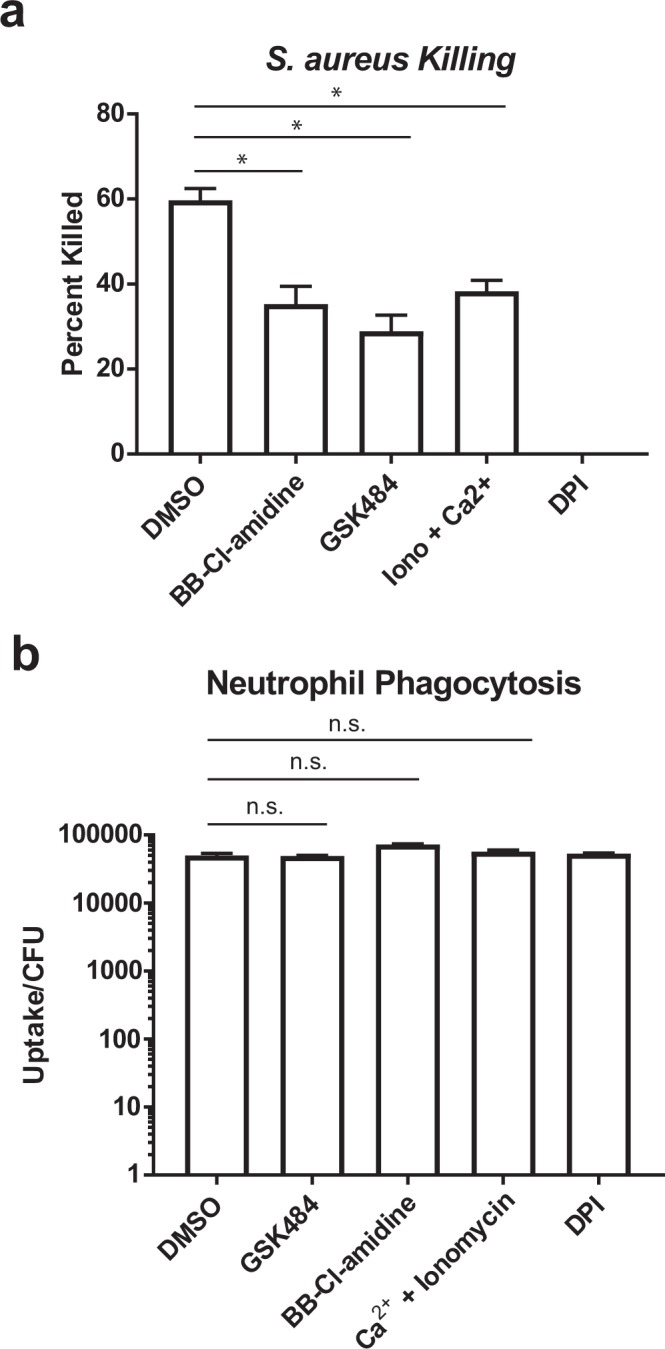
Phagocytosis and killing of *Staphylococcus aureus* by neutrophils. (**a**) *S. aureus* killing by neutrophils treated with PAD4 inhibitors, ionomycin/calcium, and DPI, as indicated. (**b**) *S. aureus* uptake by neutrophils in the same treatment groups as panel a. The data shown represent the results of four independent experiments. * denotes *p* < 0.05 and n.s. denotes *p* > 0.05.

To place our findings in a more physiologically relevant context, we tested whether disruption of the association of PAD4 with the oxidative burst machinery would affect phagocytosis and killing of live *Staphylococcus aureus*. We found that neutrophils treated with BB-Cl-amidine, GSK484, or ionomycin/calcium for 15 min significantly reduced the killing of live *S. aureus* measured at 1 h after addition of the bacteria (Fig. 6a), but had no effects at all on the uptake of bacteria by phagocytosis measured at 30 min (Fig. 6b). These data support the notion that PAD4 association with NCF proteins is important for effective killing of bacteria by human neutrophils.

## Discussion

**Figure 7:**
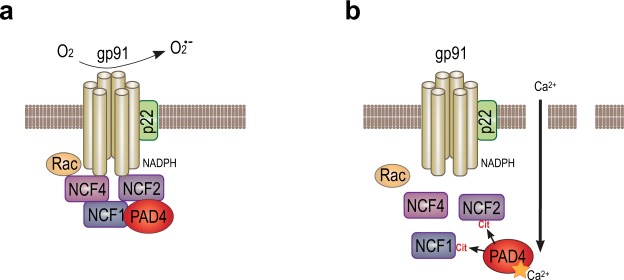
Schematic illustration of the PAD4-NCF association and its role in the oxidative burst. (**a**) Activated NADPH oxidase producing ROS in a healthy neutrophil. (**b**) Following membranolytic insults, such as perforin or MAC, high levels of Ca^2+^ influx activates PAD4 to citrullinate NCF proteins, leading to their dissociation and inhibited ROS production.

In this study, we investigated how PAD4 impacts ROS generation and bacterial killing. Our data show that PAD4 is physically associated with the cytosolic subunits of NADPH oxidase in resting neutrophils. This association increases and is essential for the assembly of active NADPH oxidase and ROS production following stimulation. The association does not require citrullination, rather citrullination leads to dissociation of the complexes (Fig. 7), suppression of NADPH oxidase assembly, ROS production, and impaired killing of phagocytized bacteria.

Our finding is the first evidence of a direct interaction between two central neutrophil enzymes in innate immunity and human disease: PAD4 and NADPH oxidase. Citrullination by PAD4 in neutrophils has gained recent attention because it appears to be responsible for the generation of modified autoantigens that drive autoimmunity in RA^16,17,22,23^. There is also a lot of interest in the role of citrullination in NET formation, which may play a role in the initiation of multiple diseases associated with extracellular DNA. However, the physiological functions of PADs remain largely unclear. Our discovery of a PAD4 - NADPH oxidase association links PAD4 to ROS production, which is critical for neutrophil function. ROS is indispensable for phagocytosis killing. With fewer mitochondria than other leukocytes^59^, NADPH oxidase is the critical source of ROS production in neutrophils.

Dysregulation of NADPH oxidase activity has been associated with autoimmunity^54–57,60^. Recently, a missense SNP variation in NCF1 was found to be associated with several autoimmune conditions. Interestingly, this SNP converts R90 to histidine and reduces ROS production^56^. It is therefore very interesting that R90 is one of the residues we find to be citrullinated in NCF1 after ionomycin treatment (Table 1). Elevated levels of NCF1 citrullination were also observed in synovial fluid from RA patients^24,25^. Since disruption of PAD4 - NADPH oxidase association by citrullination abolishes ROS production, this raises the possibility that NCF1 SNP variants may also impact PAD4 interaction, reduce NADPH oxidase activity and influence autoimmunity.

We recently reported that a portion of neutrophil PAD4 is accessible to extracellular anti-PAD4 antibodies, particularly after activation of neutrophils by a number of stimuli^40^. These exposed PAD4 molecules are catalytically active and able to citrullinate extracellular substrates^40^. By immunofluorescence staining, exposed PAD4 resides in concentrated spots on the surface, perhaps representing vesicles opening to the extracellular milieu. This is compatible with sites of PAD4-NADPH oxidase associations and the production of ROS into the lumen of such vesicles or extracellularly in open vesicles.

A novel aspect of our findings is that strong PAD4 activation through a lethal influx of calcium, termed ‘lethal hypercitrullination’ by Konig and Andrade^53^, has a negative influence on pro-inflammatory and anti-bacterial functions. This may partly explain the use of pore-forming toxins by pathogens skilled in immune evasion, such as Panton-Valentine leucocidin (PVL) of *Staphylococcus aureus*^61^. In fact, we have observed an intracellular citrullination response similar to that in Fig. 3 after treatment of neutrophils with *Staphylococcus aureus* (unpublished observation). Lethal hypercitrullination has also been suggested as a mechanism for induction of excessive citrullination in RA patients by the pore-forming activities of perforin or the membrane-attack complex of complement produced during natural killer cell- or cytotoxic T cell-mediated killing of neutrophils^23^. The relevance of this mechanism as a source of modified autoantigens in RA is supported by the detection of many of the same proteins in ionomycin- or perforin-treated neutrophils as in the synovial fluid of RA patients, exemplified by NCF1 and vimentin.

A conundrum in PAD biology is how an enzyme that requires millimolar levels of Ca^2+^ can be enzymatically active in intact cells, where resting Ca^2+^ concentrations are less than 150 nM and even the strongest receptor-mediated signaling events raise intracellular Ca^2+^ concentrations to no more than 500 nM. One possibility is that an associated protein lowers the requirement for Ca^2+^ and/or induces some of the same conformational adjustments to the active site as Ca^2+^ binding. However, it does not appear that NCF binding results in such activation. Rather, association appears to be stable only in the absence of PAD4 activity. The dissociation induced by selective small-molecule inhibitors suggests that the catalytic pocket of PAD4 is directly involved in NCF binding. As a result, these small-molecule inhibitors not only block the catalytic function of PAD4, but also disrupt binding of substrates, including proteins that utilize PAD4 as a scaffold rather than for its enzymatic activity. We therefore predict that such drugs will have a pharmacodynamic effect on processes, such as ROS production and bacterial killing even if these events may not require citrullination. In light of these data, the interpretation of the mode of action of PAD4 inhibitors in several experimental animal models may need to be reevaluated. The use of PAD4 inhibitors for the treatment of patients with RA^62^ may also need to consider such potentially suppressive effects on bacterial defense, which may also, conversely, contribute to therapeutic utility.

## Methods

### Isolation of human neutrophils

Study protocols involving human cells were approved by the MedImmune Institutional Review Board (protocol number: 2010-001 v6.0). Blood from healthy volunteers was obtained with informed consent under MedImmune’s blood donation program, and studies using human cells were performed in accordance with the Institutional Review Board guidelines. Neutrophils were isolated from heparin-anticoagulated blood on a discontinuous Ficoll gradient as previously described^63^.

### HL60 PAD4 knockdown, over expression and differentiation

Two different lentiviral constructs containing shRNA to PAD4 were purchased from Sigma (TRCN0000437969 and TRCN0000051678). Virus was generated and concentrated using standard methods. HL60 cells were transduced with the lentivirus and selected in 1 µg/ml puromycin for 1 week.

The PAD4 open reading frame was cloned into the lentiviral backbone pCDH-EF1-T2aNeo (System Biosciences). Virus was generated and concentrated using standard methods. HL60 cells that contained the stably integrated PAD4 shRNA lentivirus TRCN0000437969 or TRCN0000051678, along with parental wildtype HL60 cells, were transduced with the lentivirus and selected in 1 mg/ml geneticin for 1 week.

HL60 cells were resuspended in Roswell Park Memorial Institute (RPMI) 1640 medium (w/L-glutamine and 10% fetal bovine serum) at a density of 3 × 10^5^ cells/ml. The differentiation agent used was 0.85% N,N-dimethylformamide (DMF). Cells were allowed to grow and differentiate for 5 days before harvesting.

### Reagents

Antibodies for detecting citrullinated histone H3 (R2 citrullination, Ab176843 and R2-8-17 citrullination, Ab5103), total histone H3 (Ab24834), NCF1, NCF2, and NCF4 were from Abcam (Cambridge, MA). Antibodies for detecting PAD4 (MABE254) and citrullination (F95) were from EMD Millipore (Billerica, MA). Antibodies for PAD IP were developed as previously described^40^. Recombinant human PAD2 and recombinant human PAD4 were generated in-house. Recombinant human histone H3, pan PAD inhibitor BB-Cl-amidine, and PAD4 inhibitor GSK484 were from Cayman Chemicals (Ann Arbor, MI). The cell fraction isolation kit was from Cell Signaling (Danvers, MA). Protein G beads, DPI, ionomycin, PMA, Hoechst 33342, HRP-labeled anti-mouse immunoglobulin G (IgG), and anti-rabbit IgG secondary antibodies were from Invitrogen (Carlsbad, CA).

### Immunoprecipitation

Total neutrophil proteins were isolated with RIPA lysis buffer. The cytoplasmic fraction, and the cell membrane and membrane organelle fraction of neutrophils were isolated using a cell fraction isolation kit. IP was performed individually with PAD4, NCF1, NCF2, and NCF4 antibodies coupled to protein G beads according to manufacturer’s protocol. Tris-glycine buffer (pH 2.8) was used for elution.

### Mass spectrometry analysis

Anti-PAD4 immunoprecipitate samples were incubated in 9 M urea, 40 mM ammonium bicarbonate and 5 mM tris(2-carboxyethyl) phosphine for 1 hour followed by reduction at 45 °C for 15 min. Cooled samples were alkylated using 40 mM iodoacetamide at room temperature for 15 min. Subsequently samples were processed using 30 kDa Amicon filter (Millipore) according to “Filter Assisted Sample Preparation” (FASP) method^64^. Samples were digested using Promega 0.5 µg of lys-C/trypsin mixture at 37 °C for 12 hrs. Samples were then acidified using 0.1% TFA and desalted using Oasis HLB µElution Plate. Dried peptides were reconstituted in 100 µl of 100 mM triethyl ammonium bicarbonate and labeled using six-plex tandem mass tag (TMT) reagents according instructions provided in Thermo Scientific kit. Labeled peptides were combined and fractionated using Oasis HLB 10 mg elution Plate using 15, 20, 22, 24, 26, 28, 30, 32, 40 and 90 percent organic solution (acetonitrile in 10 mM triethyl ammonium bicarbonate). Dried labeled peptides were analyzed on a nanoflow electrospray ionization tandem mass spectrometer (LC-MS analysis). LC-MS system comprised of an Orbitrap Fusion Tribrid^TM^ mass spectrometer and Dionex Ultimate 3000 nanoRSLC^TM^. Nanoflow LC column was packed with ReproSil-Pur^TM^ 120 C18-AQ, 2.4 um particles. Solvent gradient consisted of 0.1% formic acid with increasing levels of acetonitrile concentration (up to 80% over a period of 120 minutes. The acquisition parameters used in data dependent acquisition (DDA) of the TMT labeled peptides include: MS resolution of 120 K, scan range of 400–1600, HCD MS/MS with collision energy 35, 30 K resolution. Precursor was isolated in 1.2 ± 0.345 m/z window with dynamic exclusion of 30 seconds intervals. Raw mass spectrometry data was processed using Proteome Discoverer 2.2 (PD) (Thermo Fisher Scientific) search engine Mascot (version 2.6) against recent Uniprot human protein database. Unfragmented precursor and TMT reporter ions were removed using PD nodes. Search parameters included 5 missed arginine or lysine sites, oxidation (M) and deamidation (N, Q and R) as variable modifications. Cysteine carbamidomethyl, peptide N-terminus and lysine residue modification with TMT tags were set as fixed modifications. The mass tolerances on precursor and fragment masses were set at 20 ppm and 0.06 Da, respectively. Peptides and proteins were filtered using False Discovery Rate (FDR, below 1%) of identification, which was calculated using Percolator node^65^ in PD. Peptide citrullination was manually confirmed by inspecting the MS/MS spectra for neutral loss of isocyanic acid (-43.0058 Da).

Neutrophil lysates from four different donors with and without ionomycin treatment were digested for mass spectrometry analysis using FASP protocol as explained above. Digested peptides were individually analyzed on Orbitrap Fusion mass spectrometer without labeling under same conditions except collision energy of 30 and m/z scan range 375–1575. Resulting raw data was searched using Maxquant software version 1.6.0.16^66^ with neutral loss annotation option enabled, especially to confirm the citrullination sites.

### Western blot

Equivalent amounts of proteins were separated by gel electrophoresis on 4–12% sodium dodecyl sulfate gel and then transferred to nitrocellulose membrane. For immunoblots with the anti-citrullinated Histone H3 antibody (Ab5103), anti-citrullinated Histone H3 antibody (Ab176843), total Histone H3 (Ab24834), NCF1, NCF2, NCF4, anti-PAD2 (Ab16478), and anti-PAD4 (MABE254), the iBind (Invitrogen) system was used. F95 western blot detection of pan citrullination used methods described in an earlier paper^52^. HRP-conjugated anti-mouse IgG and anti-rabbit IgG were used as secondary antibody before enhanced chemiluminescence detection. Gel quantification was performed with ImageJ.

### Confocal microscopy

Neutrophils were plated in 96-well plates and incubated at 37 °C with media, BB-CL-amidine, GSK, or ionomycin/calcium for 15 min. PMA was then added and incubated for 15 min before the plate was moved onto ice. Cells were fixed and permeabilized using Cytofix/Cytoperm (BD BioSciences, San Jose, CA) following the manufacturer’s instructions. After washing, cells were incubated with anti-PAD4-AF488 and anti-gp91-phox-AF647 (Santa Cruz, Dallas, TX), or anti-PAD4-AF488 and anti-p47-phox-AF647 (Lifespan, Seattle, WA), or anti-gp91-phox-AF488 (Santa Cruz, Dallas, TX) and anti-p47-phox-AF647 APC for 30 min on ice in the dark. Cells were washed and seeded onto a slide using a cytospin centrifuge. ProLong Gold Antifade Mountant with DAPI (Thermo Fisher Scientific, Waltham, MA) was then applied to the seeded cells and mounted with a coverslip. Images were acquired on a Leica SP5 broadband confocal microscope using a 63 × x oil objective and analyzed with Imaris imaging software (Bitplane, Belfast, UK).

### ROS production measurement

ROS production by NADPH oxidase was measured by cell oxygen consumption with Seahorse XF analyzer (Agilent, Santa Clara, CA) as previously described^67^. In brief, 96,000 cells were seeded in each well of the XF96 well plate in 160 µL of RPMI with glucose and 2 mM calcium. Cells were spun down at 200 × *g* for 1 min without a break, then equilibrated in a 37 °C incubator for 30 min. After equilibrium, the plate was loaded into an XF analyzer. Injection of buffer, ionomycin, BB-Cl-amidine, GSK484, DPI, and PMA was completed by XF analyzer. Real-time oxygen consumption rate was measured and recorded. All OCR traces were analyzed for each representative donor with 6–8 replicate wells. Data are presented as mean ± SEM.

### Bacterial phagocytosis and killing assays

Human peripheral blood neutrophils were plated (200,000 cells/96 wells). Neutrophils were incubated with GSK484 (25 µM), BB-Cl-amidine (10 µM), ionomycin/calcium (1 µM/2 mM), DPI (10 µM) for 15 min before adding bacteria. *S. aureus* (SF8300, USA300 clinical isolate) was opsonized with 10% human serum for 30 min before the experiment. Cells were washed with antibiotic-free media and incubated with *S. aureus* (MOI-0.1). Bacterial killing was determined by enumerating bacterial colony-forming units (CFU) 1 h after starting the experiment by serial dilution of the entire contents of the well (cells and media). Percent killed was calculated by subtracting the number of recovered bacteria from the inocula and dividing that number by the inocula. To monitor phagocytosis, 30 min after addition of bacteria to the neutrophil plate, the media was replaced with RPMI containing gentamycin (300 µg/ml) and the plates were placed on ice to slow the killing of internalized bacteria. The plates were incubated for an additional 30 min, at which point the cells were washed in PBS, lysed with Triton X-100, and internalized bacterial CFU were enumerated by serial dilution.

### Statistical analysis

Data analysis was performed with GraphPad Prism (GraphPad, La Jolla, CA). Paired *t*-test was used in statistical analysis.

## Electronic supplementary material

Dataset 1–3
