# Supplementary material for: Evidence for a direct link between PAD4-mediated citrullination and the oxidative burst in human neutrophils
Source: Sci Rep. 2018 Oct 15;8:15228. doi: 10.1038/s41598-018-33385-z (PMC6189209; doi:10.1038/s41598-018-33385-z)

Supplemental figures.

Evidence for a direct link between PAD4-mediated citrullination and the oxidative burst in human neutrophils

Yebin Zhou[1], Ling-Ling An[1], Raghothama Chaerkady[2], Nanette Mittereder[1], Lori Clarke[2], Taylor S. Cohen[3], Bo Chen[1], Sonja Hess[2], Gary P. Sims[1,#*] & Tomas Mustelin[1,4,#*]

Supplemental Fig. S1.

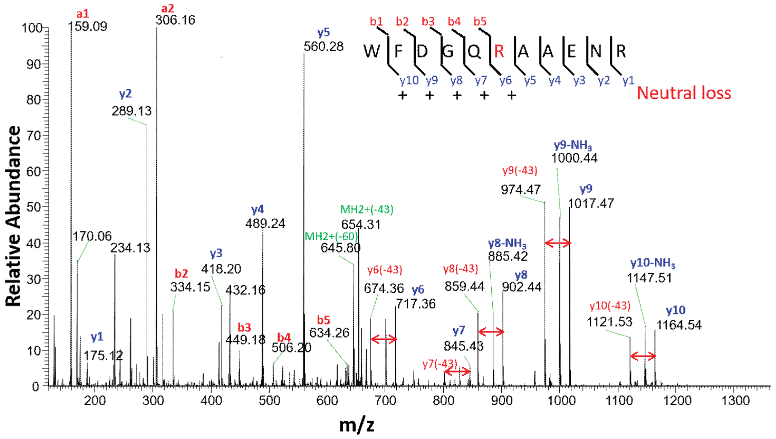

Presence of neutral loss peaks for y6 to y10 ions confirms R6 citrullination

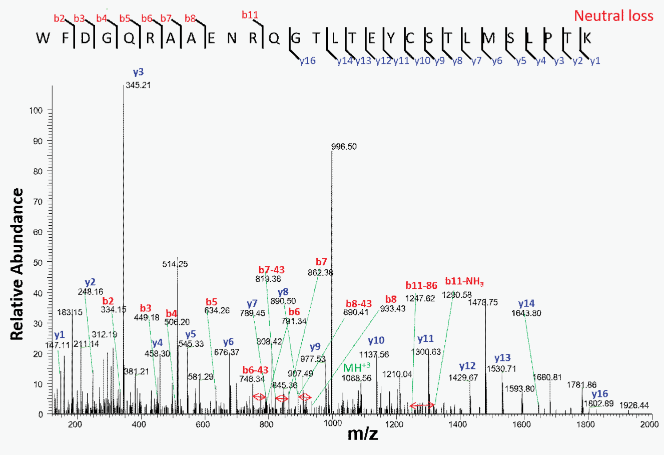

Presence of neutral loss peaks for b6 to b8 confirms R6 citrullination and neutral loss peak for b11 ions confirms R11 citrullination

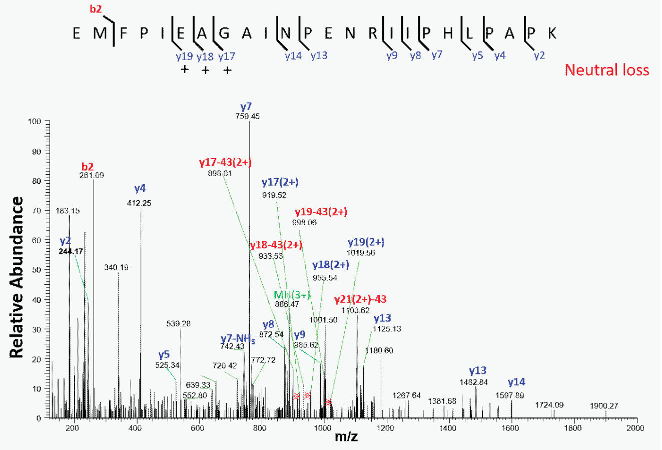

Presence of neutral loss peaks for y17 to y19 confirms R15 citrullination

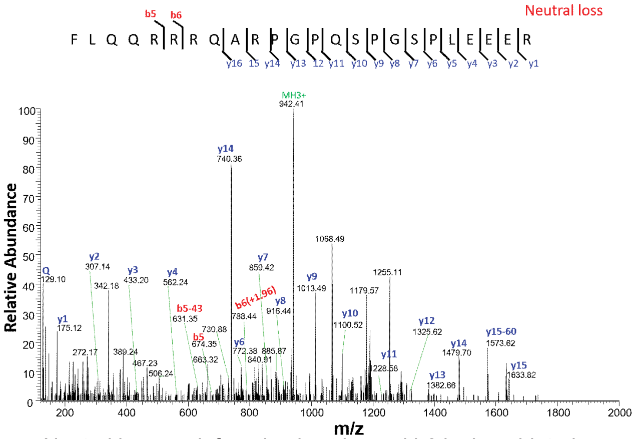

Neutral loss peak found only at b5 and b6 is deamidated indicating R5 and R6 citrullination

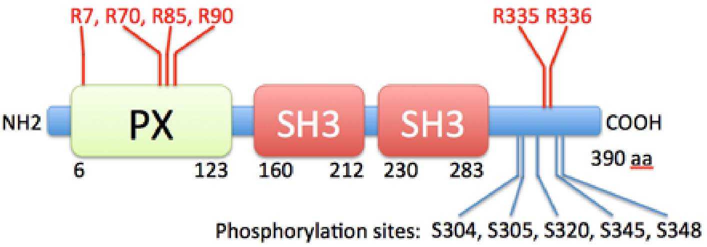

Supplemental Fig. S2.

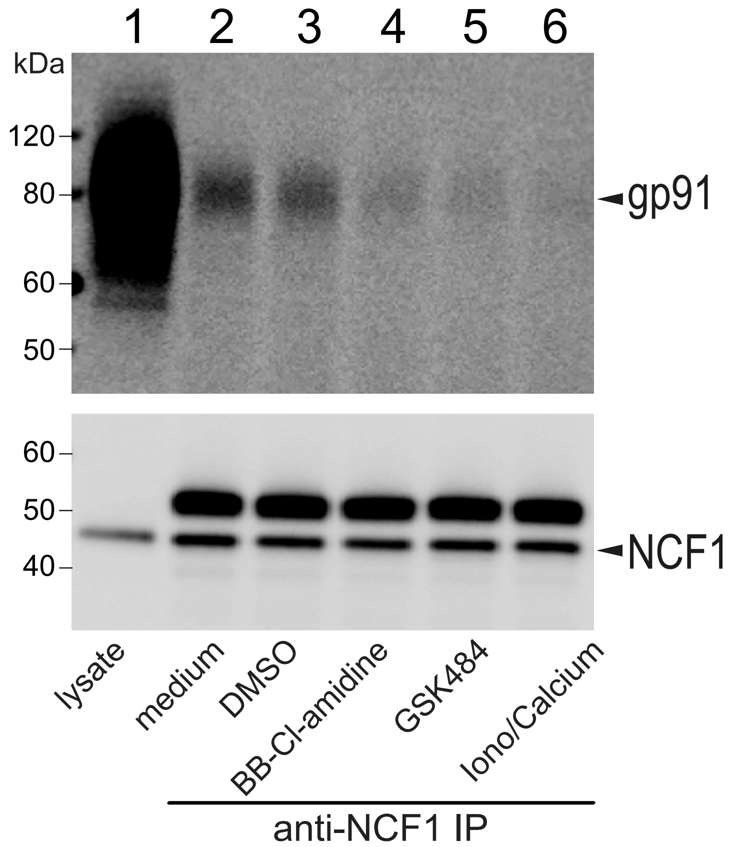

Supplemental Fig. S3.

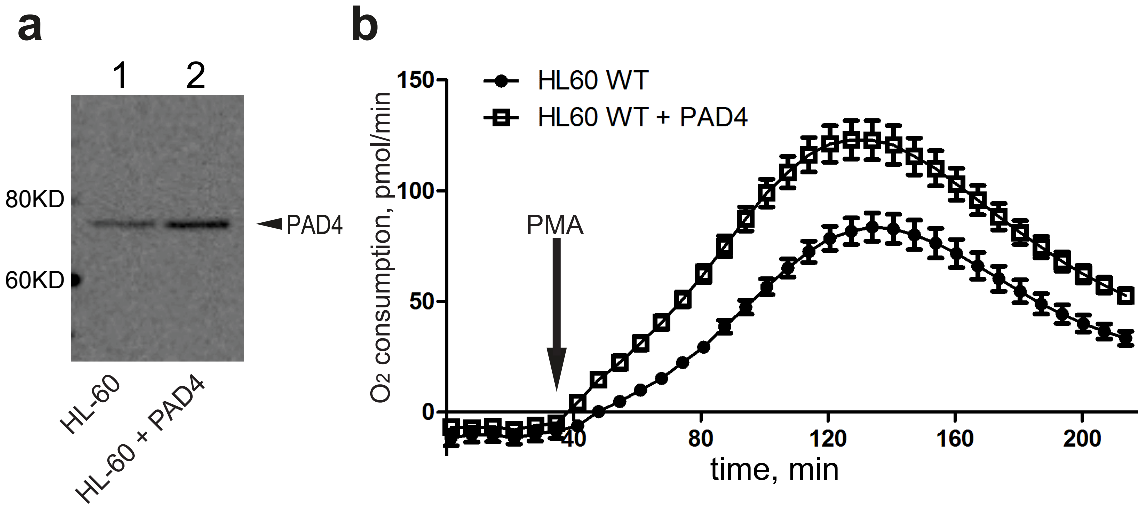

Supplement: Supplementary file 1 — Dataset 1–3 [file 41598_2018_33385_MOESM1_ESM.pdf]
